# Advancing Noninvasive Therapeutic Drug Monitoring via a 3D Microstructured Aptasensing Platform

**DOI:** 10.1021/acsomega.5c02245

**Published:** 2025-08-06

**Authors:** Hedieh Haji-Hashemi, Saeed Bahadorikhalili, Beatriz Prieto-Simón

**Affiliations:** 1 Institute of Chemical Research of Catalonia, The Barcelona Institute of Science and Technology, Av. Països Catalans, 16, Tarragona 43007, Spain; 2 Department of Electronic Engineering, Universitat Rovira i Virgili, Tarragona 43007, Spain; 3 ICREA, Pg. Lluís Companys 23, Barcelona 08010, Spain

## Abstract

Therapeutic drug monitoring (TDM) typically involves inconvenient invasive blood sampling. Sweat has been identified as an alternative biofluid that offers a convenient, noninvasive solution for real-time monitoring, supporting the growing demand for personalized healthcare. To advance in noninvasive TDM, we have delivered a novel electrochemical aptamer-based (EAB) sensing platform for sweat analysis. The sensor was built on gold-coated 3D microstructured electrodes (MSEs), fabricated via polymeric replica using macroporous silicon (macro-pSi) molds. This novel platform showed strong potential to address major challenges in sweat sensing such as the accurate and precise detection of the low analyte concentrations present in sweat, enabled by boosting the signal output thanks to the increased surface area of MSEs when compared to planar electrodes, and compliance with comfortable long-term wear, ensured by the use of flexible poly­(dimethylsiloxane) (PDMS) for MSE fabrication. As a proof of concept, we demonstrated real-time quantification of vancomycin, a narrow therapeutic window antibiotic, in artificial sweat. The MSE EAB sensor achieved up to a 2-fold increase in current and a 3-fold enhancement in signal gain compared to planar electrodes, enabling rapid (<2 min), regenerable (up to 10 times without signal loss), and precise (%RSD < 5%) quantification of vancomycin across a concentration range of 1–50 μM. Moreover, kinetic analyses and cyclic voltammetry studies conducted before and after sensor regeneration and long-term storage confirmed that MSEs preserve more effectively the aptamer probes, minimizing their potential loss and demonstrating superior stability and sensing performance compared to planar electrodes. These attributes make the sensor ideal for real-time pharmacokinetic studies via sweat analysis, enabling precise monitoring to minimize vancomycin toxicity. This approach opens new possibilities for personalized healthcare and advances real-time TDM applications beyond traditional clinical settings.

## Graphical abstract

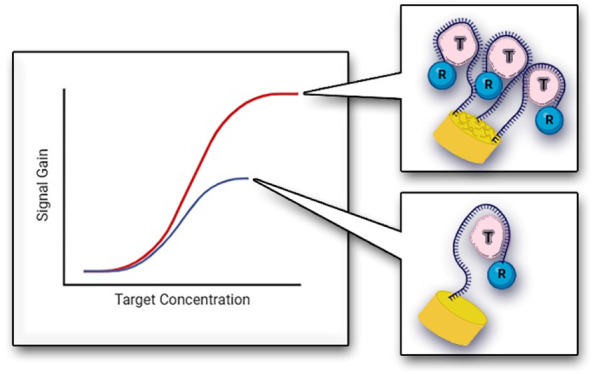

## Introduction

The paradigm shift toward personalized healthcare has intensified the need for innovative therapeutic drug monitoring (TDM) strategies. TDM, the clinical practice of measuring specific drug concentrations in biological fluids, is of paramount importance as it allows monitoring and guiding patient-specific dosage regimens, which can enhance the efficacy of drugs and avoid their toxicity.^1^ Nevertheless, standard methods for TDM involve invasive blood draws, followed by labor-intensive and high-cost laboratory-based analysis (e.g., chromatography, immunoassay), with long turnaround times, compromising the utility of TDM for optimal dosing.^2^ These limitations have sparked interest in alternative biofluids that can facilitate more convenient, accessible, and patient-friendly drug monitoring.

Sweat, a readily accessible and continuously secreted biofluid, presents an attractive noninvasive alternative to blood for real-time drug monitoring. Compared to other biofluids, sweat does not exhibit major biofouling effects, while it is rich in biological analytes, offering tremendous advantages for noninvasive and continuous monitoring.^3,4^ Moreover, recent studies have demonstrated that diverse classes of drugs, including antibiotics, antidepressants, and antihypertensives, can partition into human sweat, highlighting the feasibility of sweat-based TDM for a broad range of pharmacological treatments.^5^ This provides further evidence of sweat’s potential as a viable matrix for TDM. However, effective sweat-based sensing faces significant challenges, notably the low concentrations of target drugs and the variability in sweat composition.^6,7^ Therefore, there is a critical need for the development of highly advanced sensing technologies capable of precise and reliable measurements of the low concentrations of drugs in sweat.

One of the sensing technologies that has advanced significantly and gained considerable attention in recent years is the electrochemical aptamer-based (EAB) sensing technology. EAB sensors have the capability to conduct real-time, reagent-less, and continuous molecular measurements of a wide range of targets, including antibiotics, chemotherapeutics, drugs of abuse, proteins, etc., irrespective of the target analyte’s chemical properties.^8,9^ EAB sensors are composed of a short, *in vitro*-selected oligonucleotide (aptamer probe) as the recognition element, which is specific to the target. The aptamer is covalently attached to an interrogating electrode at one end, and at the other end, it is covalently linked to a redox reporter responsible for generating the readout signal. Target binding causes aptamer conformational changes, which alter the location and efficiency of the electron transfer of the redox reporter with the interrogated electrode, resulting in a change in electrochemical signal (Figure 1A).^10,11^ The electrochemical signal of the redox reporter is usually monitored using square wave voltammetry (SWV), where the frequency can control the magnitude and even the sign of the signal change, enabling calibration-free sensing.^12,13^ The conformation-driven signal transduction mechanism of EAB sensors renders them almost insensitive to nonspecific adsorption, allowing for direct deployment in biological fluids both *in vitro* and *in vivo* for extended periods.^14^ Additionally, these platforms are highly selective and support real-time (often reaching effective equilibrium within seconds) quantitative determination of the target concentration.^15^

**Figure 1:**
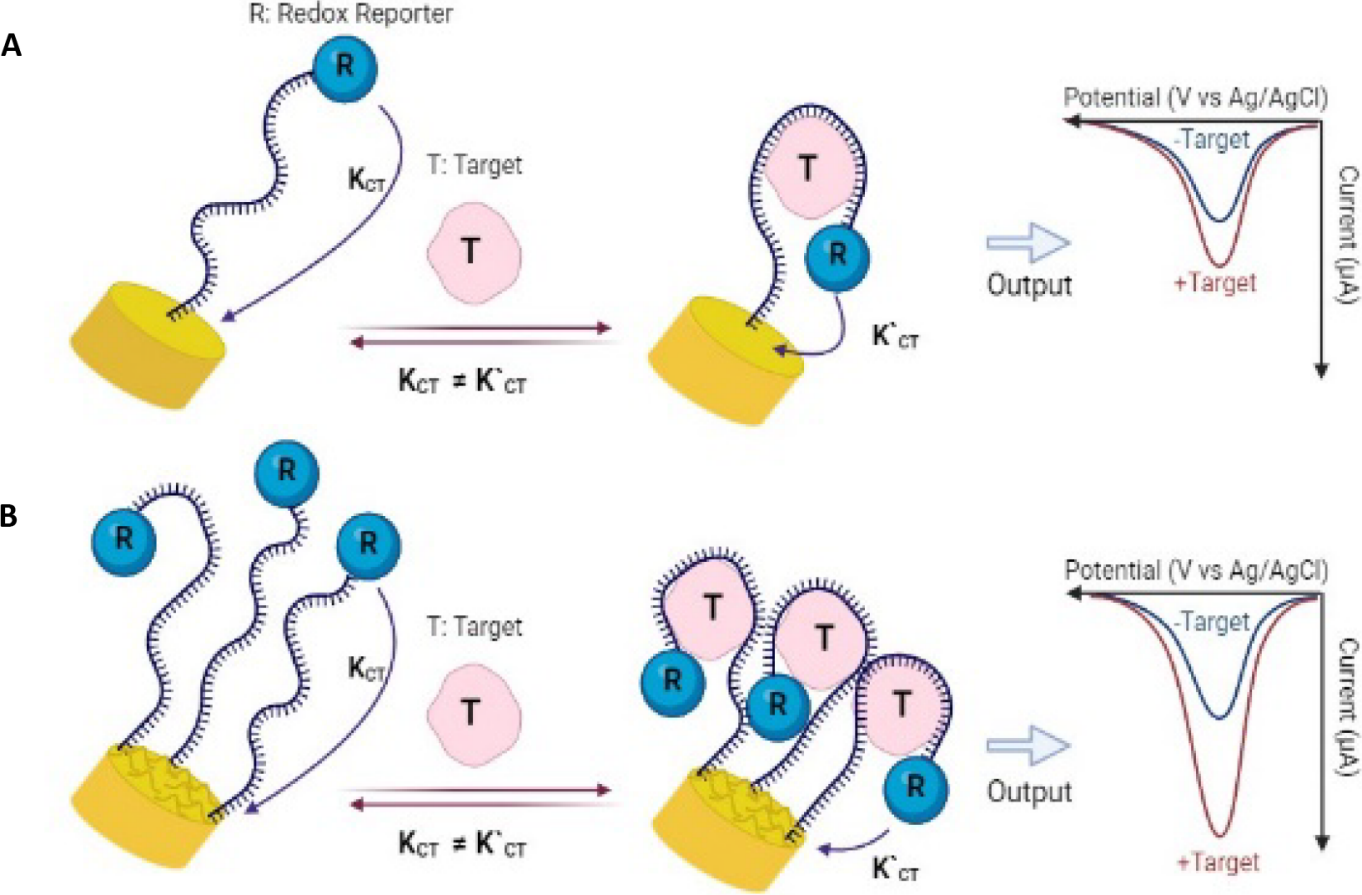
Schematic illustration of the electrochemical aptamer-based sensing approach using (A) planar and (B) MSEs.

Notwithstanding the success of EAB sensors, a limitation of this platform is its relatively low signaling output, attributed to the restricted macroscopic surface area of the electrode and the limited number of electrons each redox reporter can provide (the commonly used methylene blue reporter involves a two-electron redox process).^16^ This discrepancy is particularly striking when compared to enzymatic sensors (e.g., continuous glucose sensor), where each receptor catalytically turns over multiple copies of the target, resulting in a greater number of electrons produced per enzyme.^17^ To overcome this limitation, certain studies have explored strategies to enhance the microscopic surface area of gold electrodes, thereby improving the signaling of EAB sensors.^16,18,19^ For instance, Arroyo-Currás et al. demonstrated that creating high surface area electrodes through electrochemical roughening of gold electrodes not only enhances signaling but also improves the signal-to-noise (S/N) ratio in EAB sensors, thereby significantly enhancing the precision of *in vivo* measurements.^19^ Another study by Li et al. showcased the use of shrink-induced, wrinkled gold film substrates that effectively enhances the microscopic surface area of sensing electrodes, consequently boosting signal gain in EAB sensors.^16^ Similarly, Kumakli et al. reported that EAB sensing was enabled by electrodepositing gold dendritic structures onto microelectrodes, while unmodified microelectrodes failed to produce any measurable signal. By tuning the electrodeposition conditions, up to a 400-fold increase in surface area could be achieved, leading to a significant enhancement in sensor response.^20^

Building on this, here we explore the use of gold-coated microstructured electrodes (MSEs) in the fabrication of EAB sensors. The choice of utilizing MSEs is based on three considerations. First, conductive microstructured substrates have been reported (though not employed in EAB sensing) to provide an increased surface area compared to planar electrodes, thus enhancing the sensing signal.^21–23^ Second, a recent study demonstrated the successful application of microstructured platforms for enzymatic sensing of glucose in sweat, confirming the high potential of these platforms in hindering the loss of recognition elements from the electrode surface caused by friction between the skin and the sensing platform, which is one of the main challenges in the fabrication of wearable biosensors.^24^ Third, the flexible and soft nature of poly­(dimethylsiloxane) (PDMS) used to fabricate MSEs, coupled with its unique properties, including but not limited to biocompatibility, chemical and thermal stability, and ease of fabrication collectively render it a suitable candidate for the advancement of wearable biosensors. These characteristics not only facilitate comfortable integration on the body but also ensure the long-term stability, longevity and reliability of the biosensing devices.^25^

The synergy of employing EAB sensing and harnessing the advantages of sweat-based sensing holds significant potential, offering a pathway to advancements in the development of sweat-based wearables for noninvasive health monitoring applications. In this study, as a proof of concept, we employed MSEs for real-time quantification of vancomycin in artificial sweat using EAB sensors. This is supported by recent studies reporting the presence of certain antibiotics (e.g., vancomycin, flucloxacillin, imipenem, cefepime) in sweat, and thus confirming sweat’s value as biofluid for TDM.^26,27^ The possibility to detect vancomycin in sweat is especially relevant as this is an antibiotic with a narrow therapeutic window and significant potential for toxicity; therefore, precise control of its levels through sweat monitoring could open new opportunities for personalized therapies.^26,28^ This study lays the groundwork for the development of more efficient and reliable sensing approaches for continuous, noninvasive monitoring of key analytes in sweat, thereby advancing TDM, personalized medicine, and disease management.

## Experimental Section

### Reagents and Materials

All chemicals were purchased from Sigma-Aldrich Co. and used as received, unless otherwise noted. Vancomycin hydrochloride was obtained from Reig Jofre laboratory, Spain. All aqueous solutions were prepared using Milli-Q water (from a Milli-Q Direct purification system, resistivity = 18 MΩ). Phosphate buffered saline tablets were used to prepare a 10 mM phosphate buffer solution containing 137 mM sodium chloride and 2.7 mM potassium chloride (PBS, pH 7.4). Artificial sweat was prepared according to the reference test method EN 1811:2011 by mixing urea (0.1 wt %), sodium chloride (0.5 wt %) and lactic acid (0.1 wt %) in Milli-Q water. The pH of the solution was adjusted to a final value of 6.5 ± 0.05, using XS pH 8-PRO Basic - Bench pH Meter, XS instruments, Italy.

An HPLC-purified 3′-methylene blue- and 5′-thiol-modified oligonucleotide with affinity toward vancomycin was purchased from Biosearch Technologies (UK), with the sequence: 5′–SH–(CH_2_)_6_-CGAGGGTACCGCAATAGTACTTA TTGTTCGCCTATTGTGGGTCGG-methylene blue-3′.^29^ Upon receipt, this oligonucleotide was dissolved in TE buffer (10 mM Tris, 0.1 mM EDTA, pH 8, obtained from Integrated DNA Technologies, Inc., Belgium) at a concentration of 100 μM and then aliquoted and stored at –20 °C. The final concentration of the oligonucleotides was confirmed using a Beckman Coulter LAMBDA 1050+ UV/vis/NIR Spectrophotometer (PerkinElmer Inc., UK) using a 700 μL quartz cuvette and measuring the relative absorbance at 260 nm.

### Gold-Coated Microstructured Electrode (MSE) Fabrication

PDMS microstructures were fabricated via polymeric replica using macroporous silicon (macro-pSi) molds. Macro-pSi samples were fabricated by anodic etching of p-type (100)-oriented Si wafers with 10–20 Ohm·cm resistivity and 280 ± 20 μm thickness (Siltronix, Inc., France). Prior to etching, Si wafers were cut in 20 × 20 mm^2^ pieces and immersed in a 0.5% HF solution in absolute ethanol for 2 min at room temperature to remove the native oxide, followed by immersion in Milli-Q water for 3 min. Afterward, the samples were washed with absolute ethanol and dried with a nitrogen stream. A two-step etching process was used for the fabrication of macro-pSi molds. The first step of the etching was performed in a 1:7.5 (v/v) solution of 48% HF and dimethylformamide (DMF) using a current density of 3 mA·cm^–2^ for 1 h. The second step of etching was performed in a 1:10 (v/v) solution of 40% HF and DMF using a current density of 4 mA·cm^–2^ for 10 min. The fabricated macro-pSi was washed with absolute ethanol, dried with a nitrogen stream and then was thermally oxidized in a tube furnace at 800 °C in air for 4 h. For the fabrication of PDMS microstructures via polymeric replica using macro-pSi molds, the molds were placed in a vacuum chamber, equipped with a needle for the addition of silicone elastomer precursors (Dow Corning, 184 Silicone elastomer kit, 10:1 by weight). After the addition of silicone elastomer precursors, the samples were removed from the vacuum and cured at 110 °C for 3 h. The fabricated PDMS microstructures were mechanically peeled off the substrate. In order to make the samples conductive and ready for electrochemical biosensing applications, PDMS microstructures were uniformly coated by sputtering of 10 nm of titanium and 100 nm of gold using an RF-DC magnetron sputtering system. The deposition chamber was filled with high purity argon using a flow rate of 20 sccm, and the sputtering power applied for the deposition of titanium and gold was 150 and 100 W, respectively.

### Scanning Electron Microscopy (SEM) Image Acquisition

To analyze the morphological properties of macro-pSi, SEM was employed. Top surface and cross-section images were obtained using a Quanta 600 microscope (FEI), operating at an accelerating voltage of 20 kV. Both Everhart-Thornley (ETD) and backscattered electron (BSED) detectors were utilized for image acquisition, with 16-bit depth and a resolution of 1534 × 1024 pixels.

### Electrochemical Cell

Electrochemical measurements were performed at room temperature using an IVIUM CompactStat potentiostat (Ivium Technologies, Netherlands), along with an in-house built Teflon cell. The setup included a three-electrode cell system containing a platinum counter electrode, an Ag/AgCl reference electrode (TianjinAida Co., China), and either planar gold or gold-coated MSEs as the working electrode. Planar gold electrodes were 1.5 × 2.5 cm^2^ gold-coated microscope slides, consisting of 12.5 nm of chromium and 150 nm of gold, purchased from Telic Company (Santa Clarita, CA, USA). The geometric area of the working electrode in contact with the measurement solution was limited by an O-ring to 0.5 cm^2^ (Figure S1).

### EAB Sensor Fabrication

For EAB sensor fabrication, both planar gold and gold-coated MSEs were first electrochemically cleaned by cycling electrode potential between −0.1 and 1.6 V versus Ag/AgCl reference electrode with a scan rate of 0.1 V·s^–1^ for 10 scans in a 0.5 M sulfuric acid solution. Then the electrodes were rinsed with Milli-Q water and dried using nitrogen gas. For aptamer immobilization, an aliquot of the probe sequence was thawed and then reduced for 1 h at room temperature with a 1000-fold molar excess of tris­(2-carboxyethyl)­phosphine (TCEP) in the dark (the manufacturer provides the DNA constructs in their oxidized form, which do not effectively bind to the gold surface, so the disulfide bond must be reduced before self-assembling). Then the solution was diluted to a final concentration of 1 μM using PBS. The cleaned gold electrodes were then incubated in this solution for 2 h. Following this, the electrode surface was rinsed with Milli-Q water and incubated overnight at room temperature in a 1 mM solution of 6-mercaptohexanol (MCH) freshly prepared in PBS. Following a final rinse with Milli-Q water, the sensors were ready for use.

### Determination of Electrode Surface Area

The electroactive surface area of the gold-coated MSEs and planar gold electrodes was determined using cyclic voltammetry in a 0.05 M sulfuric acid solution. The potential applied to the electrodes was scanned from −0.1 to 1.6 V to induce the adsorption of oxygen onto the gold in a monatomic layer with a one-to-one correspondence with surface metal atoms. Upon scanning the potential back to −0.1 V, the oxygen monolayer is reduced, and the integration of the area under the reduction current gives the charge associated with the reduction of the monolayer. The charge was divided by 400 μC·cm^–2^, the charge density corresponding to a complete monolayer of chemisorbed oxygen on gold, to obtain surface area in cm^2^.^30^

### Determination of Packing Density

To determine the aptamer packing density of the prepared EAB sensors, the potential was linearly scanned from 0.0 to −0.4 V in PBS at a scan rate of 100 mV·s^–1^ to completely reduce all methylene blue tags present on the sensor surface. The area under the reduction current peak was integrated, and the total charge of reporter-modified receptors was obtained by dividing it over the scan rate. This charge value was then converted to moles and divided by the corresponding electrode surface area to obtain the packing density in picomol·cm^–2^.

### EAB Sensor Electrochemical Response Measurement and Analysis

SWV measurements were performed in a potential window of −0.1 to −0.45 V, using an amplitude of 0.025 V, a potential step of 0.003 V, and a frequency of 175 Hz (Figure S2A). The signal gain of the EAB sensors was calculated using the following eq (eq 1):

$$\mathrm{Signal}\;\mathrm{Gain}=\left(\frac{I_{\mathrm{Target}}-I_{0}}{I_{0}}\right)\times 100$$ 1

Where I_Target_ corresponds to the current in the presence of the target and I_0_ is the background current measured in the absence of the target.

For all measurements, first a set of freshly prepared sensors were interrogated at a nontarget artificial sweat solution until a stable SWV baseline signal was obtained. For the response time study, after stabilization of the baseline signal, sensors were exposed to vancomycin solutions prepared in artificial sweat at concentrations of 10, 50, and 100 μM, consecutively. During this process, SWV measurements were taken every 2 min until the signal gain stabilized in each solution. For the sensor regeneration study, signal gain was recorded after 5 min of incubation in artificial sweat, either without the target or with 50 μM vancomycin, consecutively. To plot the titration curves, as well as other results presented in this study, artificial sweat solutions spiked with known target concentrations of vancomycin were applied to the sensor surface, incubated for 5 min, and followed by SWV measurements.

## Results and Discussions

In this study, MSEs were fabricated and employed to demonstrate, for the first time, their use in EAB sensing applications. Morphological characterization of both the macro-pSi mold, used to replicate MSEs, and MSEs was performed by means of SEM. The top surface and the cross-section SEM images of macro-pSi are presented in Figures 2A and 2B, respectively. The average pore size of the macro-pSi mold is approximately 1.19 ± 0.11 μm in diameter, extending to a depth of about 9.31 ± 0.36 μm. Similarly, Figures 2C and 2D show the top surface and the cross-section SEM images of the gold-coated PDMS replica prepared from the macro-pSi mold. These SEM observations not only confirm the successful fabrication of macro-pSi but also highlight the success of the use of macro-pSi as a porous mold for the fabrication of MSEs. It is worth noting that the flexibility, lightweight nature, and user-friendly properties of PDMS ensure compliance with long-term use and facilitate comfortable integration on the body, making it an ideal candidate for advancing wearable biosensors.

**Figure 2:**
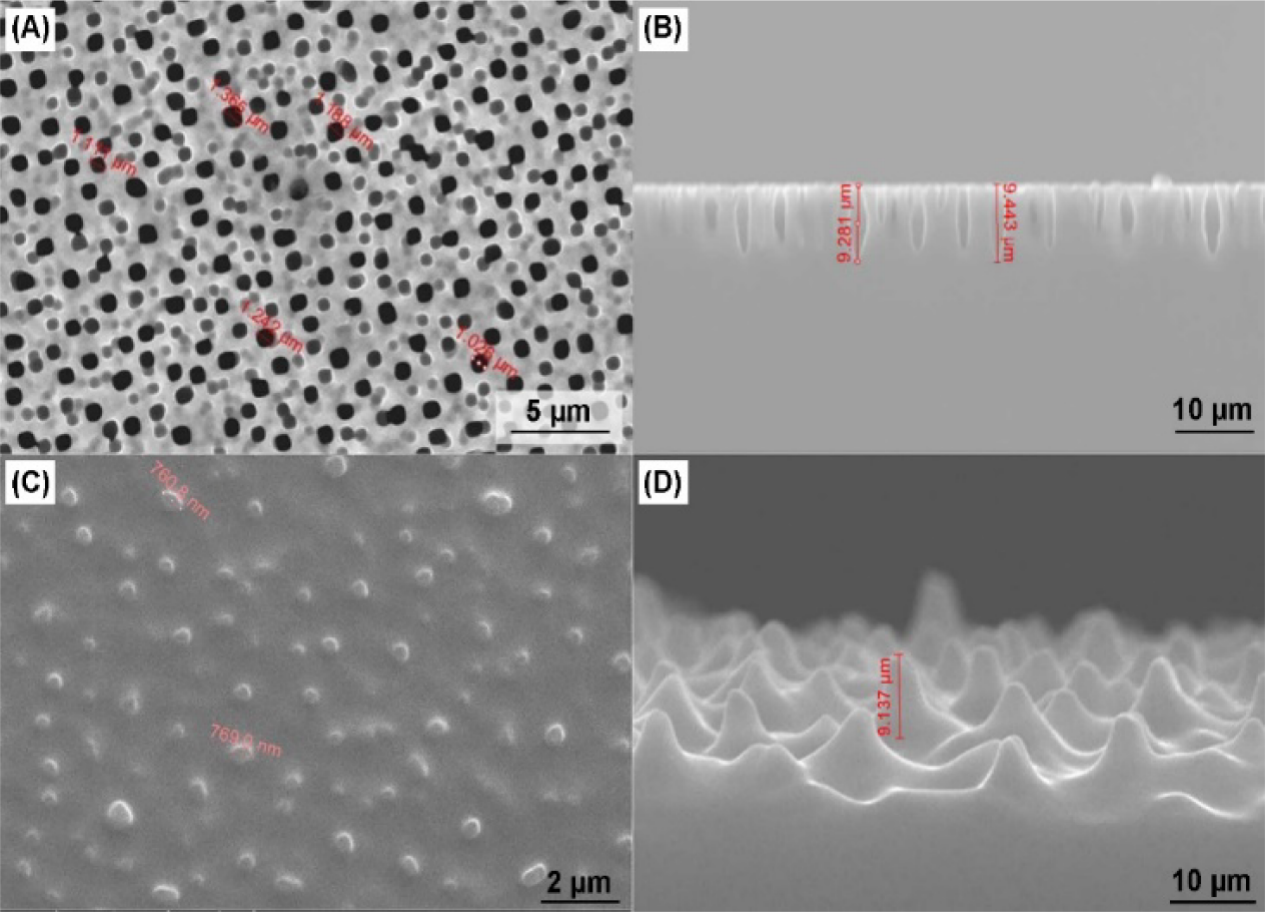
SEM images of (A) surface and (B) cross-section of macro-pSi, and (C) surface and (D) cross-section images of the gold-coated PDMS microstructure.

The microscopic surface area of MSEs was determined using cyclic voltammetry measurements (Figure S3). MSEs showed a higher reduction current at +0.9 V than planar electrodes due to the increased gold surface area afforded by their microstructured features. The active surface area of both types of electrodes was determined by the aforementioned approach, obtaining 1.46 ± 0.03 cm^2^ and 0.72 ± 0.07 cm^2^ for MSEs and planar gold electrodes, respectively.

The analytical performance of EAB sensors fabricated using MSEs was investigated, with the hypothesis that the high surface area created by 3D microstructuring will improve signaling (Figure 1B). To test this hypothesis, MSEs were compared with planar electrodes (used as control) in the fabrication of EAB sensors for the quantification of vancomycin, an antibiotic with a narrow therapeutic window and significant potential for toxicity (e.g., permanent hearing loss and nephrotoxicity).^31^ As an initial step, vancomycin EAB sensors fabricated using MSEs were challenged with increasing concentrations of vancomycin. As expected, a monotonically increase in current was observed (Figure 3A), confirming the ability of this platform in sensing the target. In comparison to the sensors fabricated using planar gold electrodes, MSE-based sensors showed a 2-fold increase in baseline current and a 3-fold increase in response (Figure 3B,C). The observed improvement in signaling with MSE sensors can be attributed to the higher surface area provided by the microstructures, which accommodates comparatively a larger number of aptamer probes, with a larger number of redox reporter molecules contributing to the signal measured. Moreover, having more aptamer probes available at the electrode surface can consequently facilitate target binding, further enhancing the response to the target. This aligns with previously reported strategies applied to increase the electroactive surface area. For instance, Kumakli et al. demonstrated that the modification of microelectrodes with gold dendritic structures significantly increased their surface area. By tuning electrodeposition conditions to optimize the dendritic structure size, a 4-fold increase in electrode surface area was reported, resulting in a 3-fold enhancement in signal gain.^20^ Similarly, Li et al. reported a wrinkled gold film approach, achieving approximately a 5.5-fold increase in surface area relative to planar substrates, leading to up to a 10-fold improvement in sensor signals for certain analytes.^16^

**Figure 3:**
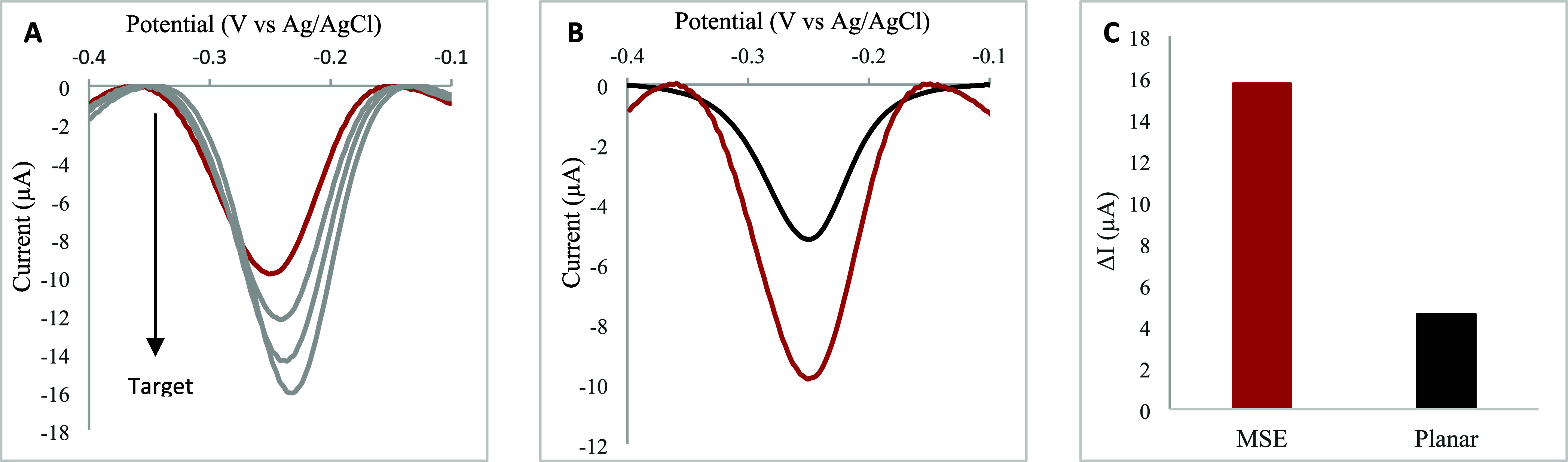
(A) Square wave voltammograms obtained for an MSE EAB sensor upon incubation in artificial sweat solution before (red) and after spiking vancomycin to 5, 10, and 25 μM (gray). (B) Baseline square wave voltammograms of MSE (red) and planar gold electrode (black) recorded in artificial sweat solution. (C) Current change for MSE and planar gold electrode EAB sensors; ΔI (μA)= I - I_0_, where I_0_ is the baseline current measured in artificial sweat solution, and I corresponds to the measured current in a 100 μM vancomycin solution.

It is noteworthy that EAB’s signal gain strongly depends on the density that aptamer probes are packed on the electrode surface,^32^ while this density is closely linked to the roughness factor of the gold electrode surface.^33^ For instance, Arroyo-Currás et al. reported that the improved gain shown by roughened electrode surfaces was mainly attributed to the high aptamer packing densities achieved under specific fabrication conditions rather than to the increased surface area alone.^19^ To explore whether aptamer packing density plays a role in the improved signaling of MSE-based sensors, we calculated the aptamer packing density of both MSE and planar sensors using the previously outlined procedure. Surprisingly, the calculated values were similar, measuring 1.09 ± 0.05 picomol·cm^–2^ and 1.07 ± 0.06 picomol·cm^–2^ for MSE and planar sensors, respectively. Therefore, the observed improvement in signaling with MSE sensors is solely attributed to the higher surface area provided by the microstructures. These results closely align with Kumakli et al.’s work, as the aptamer packing densities remained similar for electrodes with varying dendritic surface areas, corroborating our finding that the improved signaling in MSE sensors is due to the increased surface area provided by the microstructures.^20^

The EAB sensors built on both MSE and planar electrodes respond rapidly to vancomycin, reaching 90% of the total response within a couple of minutes (Figure 4A and Figure S4A). Figure 4B shows the titration curves for both MSE and planar EAB sensors. As vancomycin concentrations increased, both sensors produced the monotonic Langmuir isotherm behavior expected for this type of binding event. As expected, in comparison to the sensors fabricated using planar electrodes, a greatly improved signal gain was observed at the saturation concentration for MSE-based sensors. The titration curves were well-fitted to the Langmuir-Hill isotherm:

$$\mathrm{Signal}\;\mathrm{Gain}=\gamma \frac{\left[T\right]}{K_{D}+[T]}$$ 2

where [T] is the concentration of vancomycin, γ is the maximum signal output, and K_D_ is the apparent dissociation constant.^34^ The fits yielded R^2^ values of 0.993 for the planar sensors and 0.992 for the MSE-based sensors, with corresponding K_D_ values of 26.3 ± 3.3 μM and 46.6 ± 4.1 μM, respectively (Figure S5A,B). This behavior is consistent with the observations of Kumakli et al.^20^ and Arroyo-Currás et al.,^19^ who likewise reported higher K_D_ values for electrodes possessing greater curvature than their planar counterparts. We attribute the elevated K_D_ recorded for the MSE devices to curvature-induced shifts in the conformational-switching equilibrium of the aptamers. Specifically, we propose that the three-dimensional topography of the microstructured electrodes mitigates aptamer collapse and aggregation, thereby favoring more stable nonbinding conformations, whereas planar surfaces are more prone to these effects.^20^ By stabilizing the aptamers in a nonbinding, low-signal state, the system preserves a larger fraction of probes ready to respond upon target engagement. Although this stabilization amplifies the magnitude of the binding-induced signal, the accompanying shift in conformational equilibrium toward the nonbinding state reduces the apparent binding affinity, producing the modestly higher K_D_ values observed.

**Figure 4:**
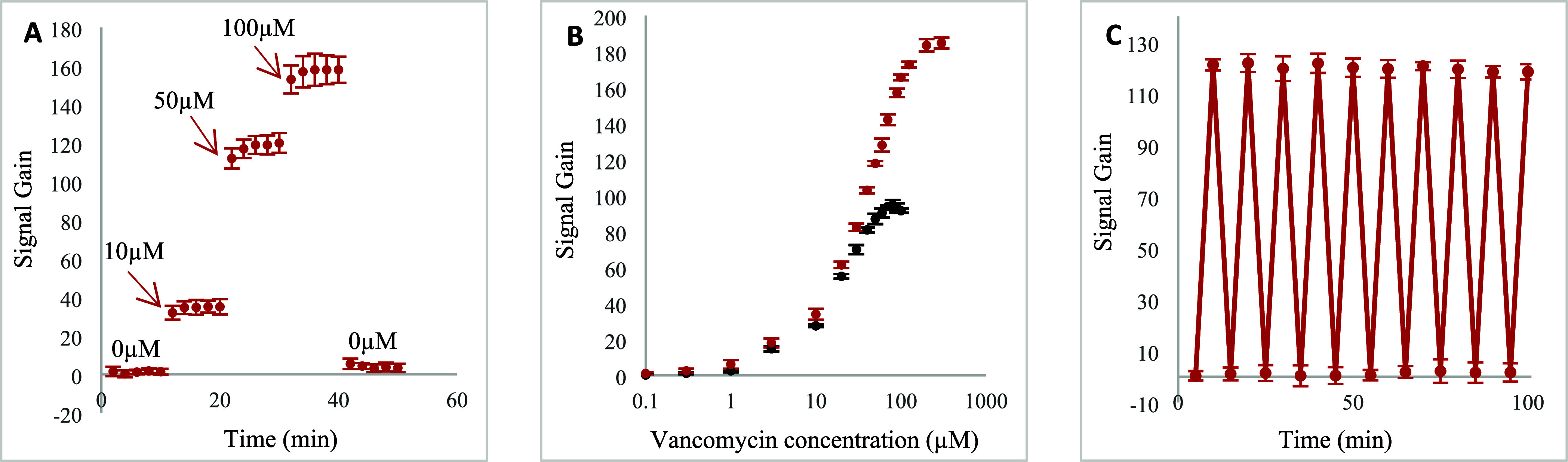
(A) MSE EAB sensor incubation time optimization; signal gain was recorded every 2 min after sensors were exposed to artificial sweat solution and vancomycin solutions prepared in artificial sweat at concentrations of 10, 50, and 100 μM. (B) Titration curves for the MSE (in red) and planar (in black) EAB sensors; signal gain was recorded consecutively, after 5 min incubation in artificial sweat solution spiked with known concentrations of vancomycin. (C) Regeneration cycles of the MSE EAB sensor; signal gain was recorded consecutively, after 5 min incubation in artificial sweat solution with no target or containing 50 μM vancomycin. The illustrated values are the average signal gain values achieved from 3 individual EAB sensors, and error bars represent the standard deviation of these measurements.

To further elucidate the performance enhancements provided by microstructured electrodes, electron-transfer kinetics were assessed by square-wave voltammetry over frequencies ranging from 5 to 700 Hz, examining both the unbound and vancomycin-bound states. Representative plots of Ip/*f* versus *f* (Figure S2C,D) and the associated kinetic analyses are provided in the Supporting Information. Compared to planar electrodes, the MSE exhibited slower electron-transfer rates (*k*_s_) in the unbound state, but faster kinetics upon target binding. These kinetic insights endorse the amplified signal gains shown by MSEs, and support the hypothesis that the microstructured surface stabilizes the aptamer in a binding-competent conformation, thereby rationalizing the slight increase observed in the apparent K_D_.

Although MSE-based sensors show a slightly lower binding affinity, this is compensated by the improved signal gain, precision, and practical detection range achieved for vancomycin monitoring. Notably, the MSE-based sensors provide a broader dynamic range (0.3–200 μM) in comparison to the planar sensors (1–90 μM), which is consistent with the enhanced signal output of microstructured electrodes.

Moreover, the practical quantitation limit (PQL) of both MSE and planar EAB sensors was determined experimentally as the lowest analyte concentration that provided acceptable precision in the calculated signal gain (RSD < 10%) based on replicate measurements. The PQL was found to be 0.3 μM and 1 μM for MSE and planar EAB sensors, respectively. The lower PQL achieved with the MSE-based EAB sensor makes it a promising tool for vancomycin TDM, with strong potential to mitigate toxicity.

Next, EAB sensors regeneration was evaluated using the previously outlined protocol. Both MSE and planar EAB sensors can be easily reused, with a 99% recovery of the baseline signal within minutes of being placed back in the artificial sweat solution (Figure 4A and Figure S4A). This rapid and high recovery can be attributed to the aptamer’s swift conformational change, a key advantage of EAB sensing platforms, enabling them to quickly reach equilibrium both in the presence and absence of the target. In terms of stability during consecutive regeneration cycles, the sensors fabricated using MSEs demonstrated slightly higher stability compared to the planar electrode-based sensors. After 10 regeneration cycles, while the planar sensors retained 85% of their initial signal gain (Figure S4B), the MSE sensors maintained an impressive 98% of their initial signal gain (Figure 4C). To understand the reason behind this enhanced stability, we recalculated the probe density after the regeneration experiments. The planar electrodes showed around 21% decrease in probe density, while the MSEs exhibited roughly 7% decrease. This notable difference indicates that MSEs preserve aptamer probes more effectively on their surfaces, minimizing the loss of biorecognition elements and confirming their superior performance and stability relative to planar electrodes. These observations are further supported by previously discussed kinetic findings (see the Supporting Information) and cyclic voltammograms recorded after regeneration cycles (Figure S6A,B), collectively indicating that the microstructured surface effectively stabilizes aptamers in a binding-competent conformation. This stabilization justifies both the increased electron-transfer kinetics upon target binding and the enhanced ability of MSEs to maintain their sensor performance across multiple regeneration cycles.

The significantly improved signaling of EAB sensors fabricated using MSEs facilitates high-precision measurements. To demonstrate this, the titration fits from the sensors (Figure 4B) were employed to estimate the recovered concentrations from another set of sensors outside the titration set. Specifically, initially, the Langmuir-Hill isotherm (eq 2) was utilized to fit the titration curves, deriving the correlation between vancomycin concentration and the signal gain. Once the fitting constants (γ and K_D_) were obtained, they were used to convert the signal gain values from the independent sensors into vancomycin concentrations.

As Table 1 shows, sensors fabricated using MSEs exhibited excellent recovery rates, with estimated values within ± 10% of the spiked concentration across the range of 0.3 μM to 50 μM. In contrast, planar sensors showed lower sensor precision, as represented by the recovery rates. The higher sensor precision in the case of MSE sensors could be attributed to their higher baseline current and higher signal gains achieved for different vancomycin concentrations. This positions the MSE EAB sensor as a promising tool to be used for the TDM of vancomycin, offering potential for mitigating its toxicity. Considering the sweat-blood correlation of vancomycin concentration (approximately 0.2–2.5%),^26^ and the recommended blood concentration below 27 μM (40 mg L^–1^) to avoid or reduce toxic effects,^35^ the MSE EAB sensor demonstrates significant potential for clinical application. It is important to highlight that this broad correlation range originates from interindividual variability. Assuming an average correlation of approximately 1.35%, the corresponding vancomycin concentration in sweat at the minimum toxic blood concentration (27 μM) would be approximately 0.35 μM, a level comfortably fitting within the detection capability of our sensor, which reliably quantifies concentrations as low as 0.3 μM.

**Table 1:** Recovery Rates of MSE and Planar EAB Sensors Detecting Vancomycin^a^

	Recovery %	
Spiked concentration (μM)	MSE EAB sensor	Planar sensor
0.3	108.1 ± 5.6	ND
1	109.3 ± 2.4	71.0 ± 6.1
3	101.8 ± 3.3	121.0 ± 4.4
10	94.0 ± 1.6	111.2 ± 2.1
20	99.0 ± 1.1	117.9 ± 1.8
30	93.6 ± 2.0	107.0 ± 2.4
40	104.1 ± 1.8	149.1 ± 2.3
50	108.8 ± 0.4	90.2 ± 3.1

a ND: Not detected.

Furthermore, the storage stability of the MSE and planar EAB sensors was investigated over a 14-day period when EAB sensors were incubated in PBS and stored at 4 °C. The signal gain of these EAB sensors tested in a 50 μM vancomycin solution prepared in artificial sweat was measured at a regular interval time of 2 days. While the signal response of the planar EAB sensors decreased by 60% after 6 days, the MSE EAB sensors maintained a stable signal response, retaining 100% of the initial gain even after 8 days. This suggests the aptamer immobilization on the MSE electrodes was robust, efficiently retaining the biorecognition layer intact during this time frame. By day 14, the signal response for the MSE and planar EAB sensors had decreased to 60% and 7% of the initial gain, respectively. Similarly to the study previously performed to support the improved operational stability of MSE EAB sensors upon several regeneration cycles, estimating here the changes in probe density of both MSE and planar gold EAB sensors after the 14-day period was used to shed light on the improved storage stability of MSE EAB sensors. The MSE sensors showed around 35% decrease in probe density, whereas the planar electrodes exhibited a decrease of more than 60%. This significant difference confirms that the MSEs better preserve the aptamer probes on the electrode surface over time, leading to enhanced storage stability and prolonged sensor lifetime. These findings are further supported by kinetic analyses (see the Supporting Information) and cyclic voltammograms recorded before and after storage (Figure S6C,D), reinforcing the conclusion that the microstructured surfaces effectively stabilize aptamers in their binding-competent conformation. This structural stability significantly extends the functionality and lifetime of the sensors during prolonged storage. This superior performance makes MSEs highly suitable for applications requiring long-term monitoring, as they outperform planar electrodes in maintaining their functionality over extended periods of time.

## Conclusions

This study opens new avenues for advancing wearable sensing technologies for TDM, emphasizing the synergy between EAB sensing and the advantages of sweat-based sensing for noninvasive molecular monitoring. The results reported in this work validate the successful implementation of gold-coated MSEs in the development of EAB sensors for the quantification of vancomycin in artificial sweat. The increased surface area provided by the microstructures significantly enhances the signal output and gain of EAB sensing, facilitating the sensitive and accurate detection of vancomycin. Specifically, the MSE-based EAB sensors demonstrated up to a 2-fold increase in current and a 3-fold increase in signal gain compared to planar gold electrodes, enabling rapid and precise quantification of vancomycin within a ± 10% error margin across the range of 0.3 μM to 50 μM, offering potential for mitigating toxic effects. These attributes position the platform as an ideal candidate for real-time pharmacokinetic studies in sweat, providing a noninvasive, reliable, and patient-friendly approach for monitoring drug levels. Moreover, the feasibility of noninvasive monitoring of other antibiotics, such as flucloxacillin, imipenem, and cefepime, underscores sweat’s value as a biofluid for TDM of antibiotics.^27^ Overall, this work not only lays the groundwork for efficient and reliable wearable biosensing in sweat but also represents a significant step forward in real-time, continuous monitoring of antibiotics, opening new possibilities for personalized healthcare and combating the emergence of antimicrobial resistance driven by the misuse of antibiotics. Future development, including the integration of this MSE-based EAB sensor into a wearable patch for sweat-based sensing, could substantially expand its impact in clinical settings, paving the way for improved TDM. Such an integration will require addressing key challenges, including efficient sweat collection, handling the inherent variability in sweat composition (e.g., pH and ionic strength), and ensuring stable on-body integration.

## Supplementary Material



## Notes

Additional experimental details and results including: photographs of the electrochemical cell setup, plots of signal gain as a function of the square-wave interrogation frequency, kinetic study of both MSE and planar EAB sensors, cyclic voltammograms of both MSE and planar gold electrode recorded in sulfuric acid, Langmuir-Hill isotherm fitting of titration curves, results from the incubation time optimization and regeneration studies of the gold planar electrode-based sensors, and cyclic voltammograms recorded before and after both regeneration cycles and long-term stability tests (PDF)

## †

Department of Petroleum Engineering, Texas A&M University, College Station, Texas 77840, United States (S.B.)
