# Supplementary material for: Advancing Noninvasive Therapeutic Drug Monitoring via a 3D Microstructured Aptasensing Platform
Source: ACS Omega. 2025 Aug 6;10(32):35689–97. doi: 10.1021/acsomega.5c02245 (PMC12368656; doi:10.1021/acsomega.5c02245)
Supplement: Supplementary file 1 [file ao5c02245_si_001.pdf]

Supporting Information

Advancing Noninvasive Therapeutic Drug Monitoring via a 3D Microstructured Aptasensing Platform

Hedieh Haji-Hashemi[1]*, Saeed Bahadorikhalili[2†], Beatriz Prieto-Simón[1,3]*

[1] Institute of Chemical Research of Catalonia, The Barcelona Institute of Science and Technology, Av. Països Catalans, 16, 43007, Tarragona, Spain

[2] Department of Electronic Engineering, Universitat Rovira i Virgili, 43007, Tarragona, Spain

[3] ICREA, Pg. Lluís Companys 23, 08010, Barcelona, Spain

Table of Contents

Figure S1……………………………………………………………………………………S2

Figure S2……………………………………………………………………………………S2

Kinetic study…………………………………………………………………………………S3

Figure S3……………………………………………………………………………………S4

Figure S4……………………………………………………………………………………S5

Figure S5……………………………………………………………………………………S6

Figure S6……………………………………………………………………………………S7

† Present Address: Department of Petroleum Engineering, Texas A&M University, College Station, TX, USA.

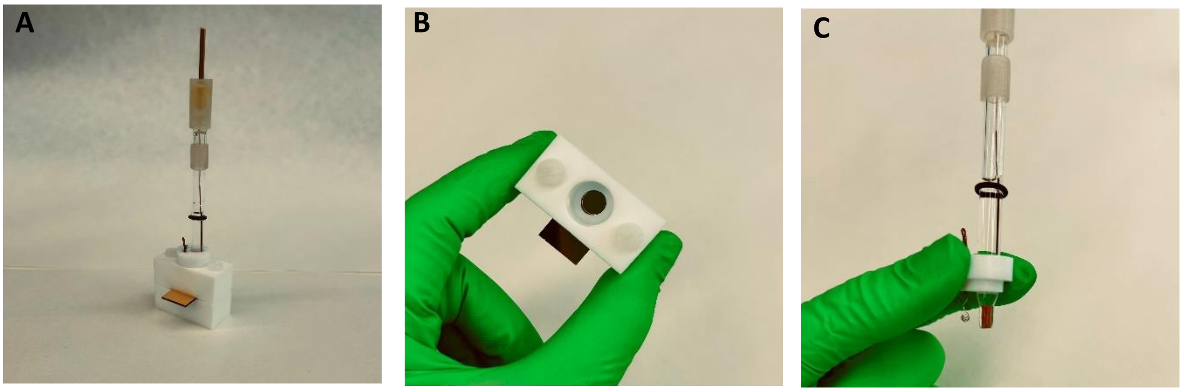

Figure S1. (A) In-house built electrochemical cell utilized for conducting all electrochemical measurements. (B) Open view of the cell showing the volume for the measuring solution, and the geometric area (0.5 cm^2) of the working electrode delimited by an O-ring. (C) Reference and platinum counter electrodes utilized for conducting electrochemical measurements.

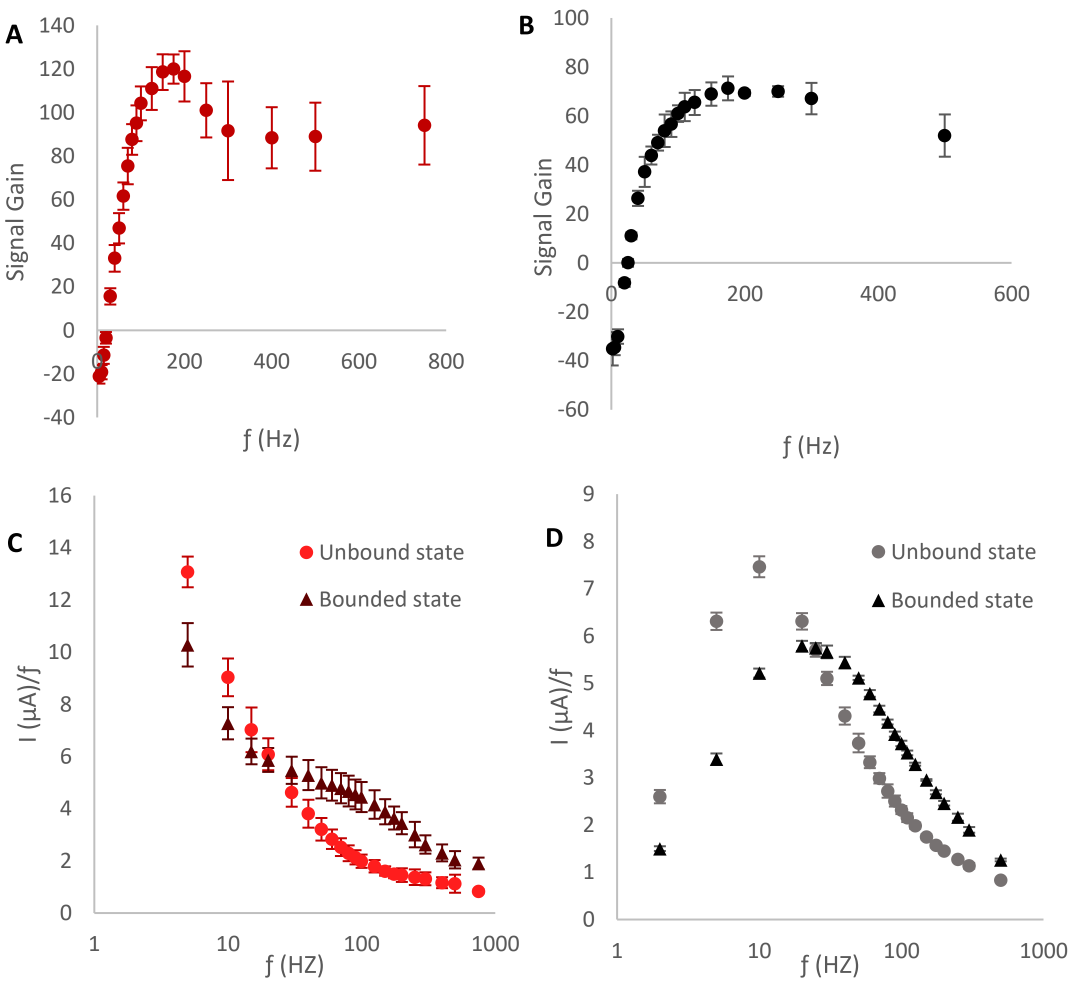

Figure S2. Plot of signal gain as a function of the square-wave interrogation frequency for the (A) MSE and (B) planar EAB sensors in response to 50 µM of vancomycin, and corresponding Ip/f versus f plots for the (C) MSE and (D) planar EAB sensors in the absence (circles) and presence (triangles) of 50 µM of vancomycin. Values are the average signal gain value achieved from 3 individual EAB sensors, and error bars represent the standard deviation of these measurements.

Kinetic Study of the MSE and Planar EAB Sensors

To gain further insights into the electrochemical performance enhancements observed with MSE, we evaluated electron-transfer kinetics using square-wave voltammetry. We have recorded square-wave voltammograms over a wide frequency range (5 – 700 Hz) for both the unbound and vancomycin-bound states, and plots of *Ip/f* versus *f* were constructed for each electrode type. In each trace the plot shows a clear maximum, and the frequency at this maximum can be converted to the electron-transfer rate constant using

$$k_s = \kappa_{max} \cdot f_{max}$$

as derived by Komorsky-Lovrić and Lovrić [1]. The proportionality factor κ_{max} depends on the transfer coefficient (α), the square-wave amplitude, and the number of electrons transferred (n), and is independent of both the step potential and the surface coverage of the redox label. Because these parameters are fixed for the methylene blue reporter used here, κ_{max} is constant, so f_{max} serves as a direct proxy for k_s.

Using this approach, two clear trends emerged:

1. **Unbound state.** The planar electrode exhibits the higher k_s indicating that the methylene-blue reporter resides, on average, closer to the electrode surface than on the MSE. This is consistent with our hypothesis that planar surfaces encourage partial collapse/aggregation of the aptamer, bringing the reporter nearer to the electrode surface even in the absence of target.

2. **Bound state**. After target binding, the situation reverses; the MSE shows the higher k_s. The larger curvature apparently allows the aptamer to complete its binding-induced conformational change without the steric constraints present on the planar surface, positioning the reporter closer and enabling faster electron transfer.

Consequently, the change in k_s upon binding (Δk_s) is much larger on the microstructured electrodes, mirroring the greater signal gain observed in Figure 4B. These results corroborate the notion that microstructure reduces aptamer collapse/aggregation in the unbound state while facilitating productive conformational switching upon target recognition, thereby explaining both the kinetic data and the enhanced analytical performance.

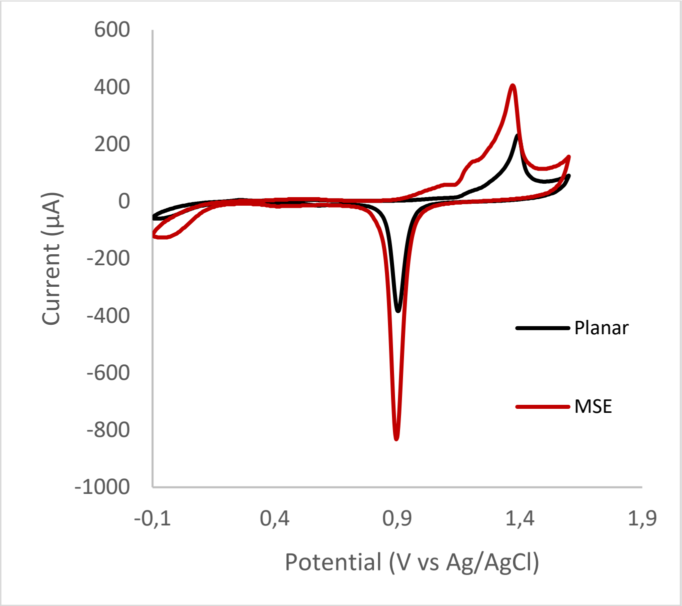

Figure S3. Cyclic voltammograms of MSE (red) and planar electrode (black) recorded by cycling electrode potential between -0.1 and 1.6 V versus Ag/AgCl reference electrode with a scan rate of 0.1 $V \cdot s^{-1}$, in a 0.05 M sulfuric acid solution.

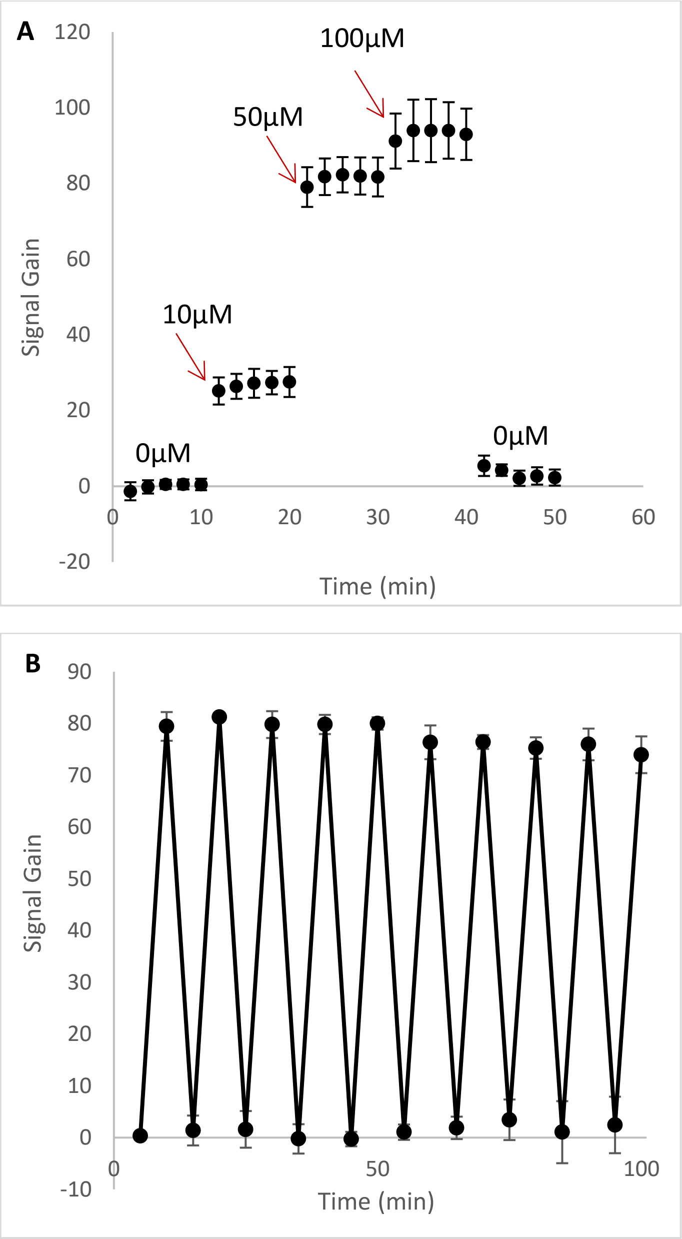

Figure S4. (A) Planar EAB sensor incubation time optimization; signal gain was recorded every 2 min after sensors were exposed to artificial sweat solution and vancomycin solutions prepared in artificial sweat at concentrations of 10, 50, and 100 μM, consecutively. (B) Regeneration cycles of the planar EAB sensor; signal gain was recorded consecutively, after 5 min incubation in artificial sweat solution with no target or containing 50 μM vancomycin. The illustrated values are the average signal gain values achieved from 3 individual EAB sensors, and error bars represent the standard deviation of these measurements.

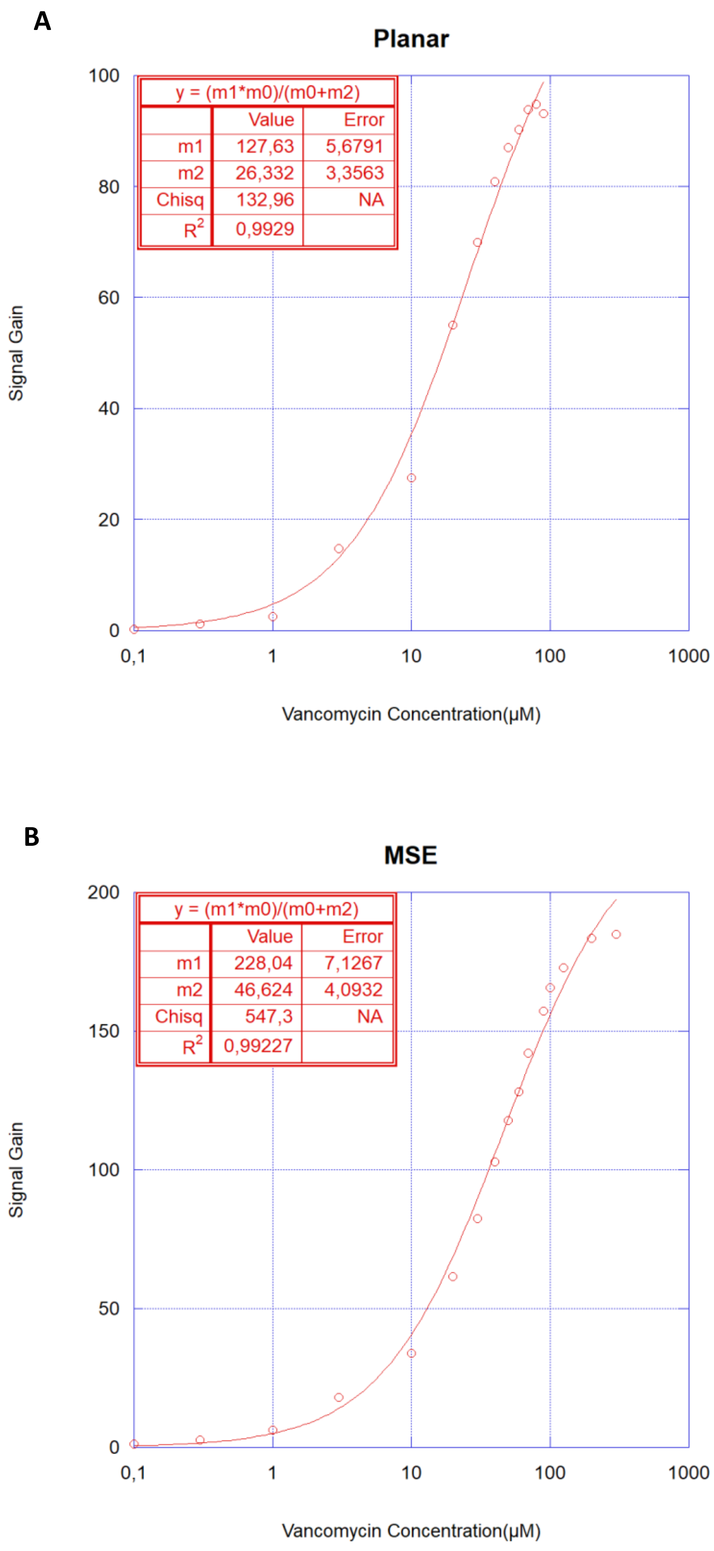

Figure S5. Langmuir-Hill isotherm fit of the obtained titration curves for (A) Planar EAB sensor and (B) MSE EAB sensor. Fitting of titration curves were performed using KaleidaGraph software.

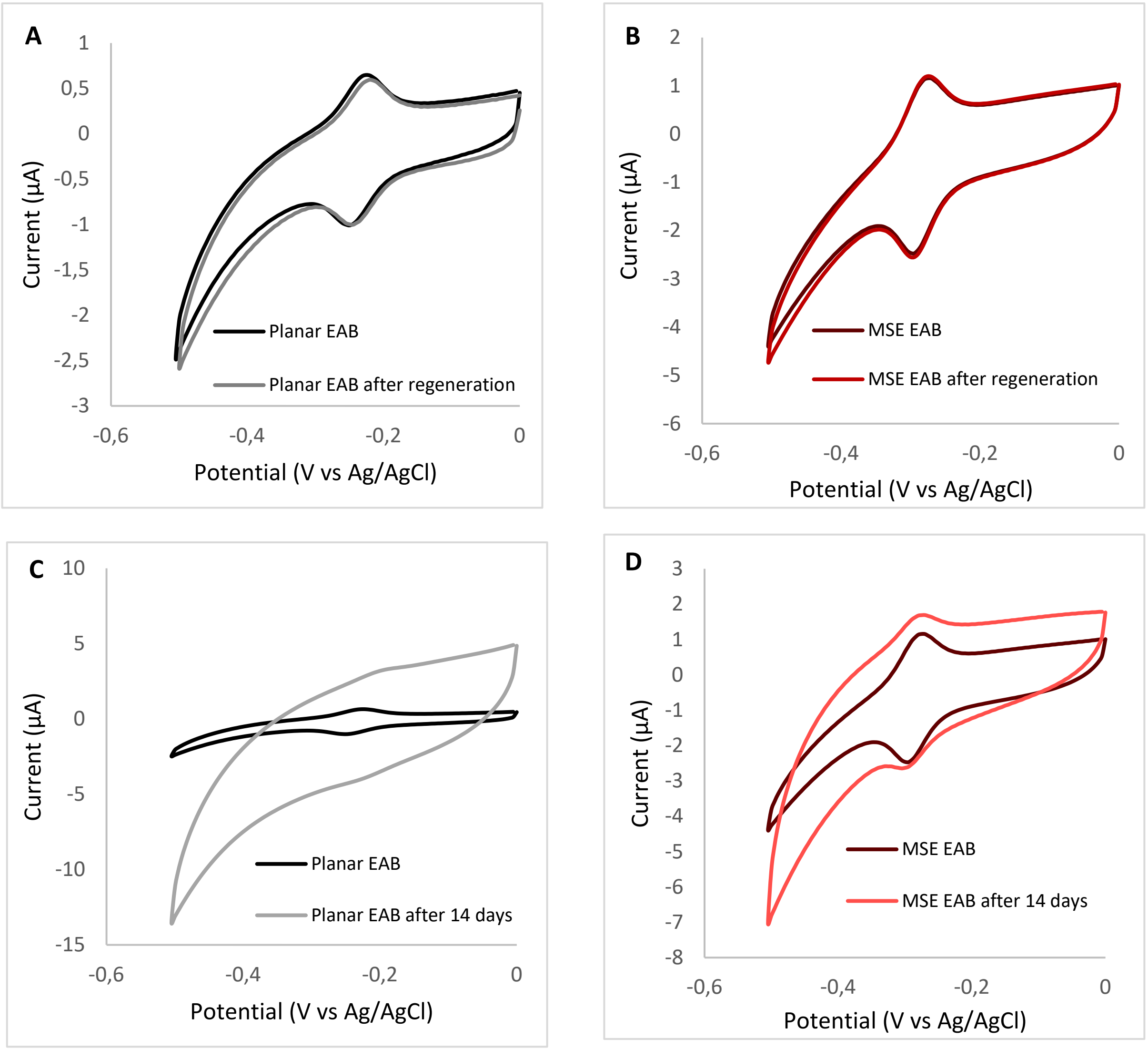

Figure S6. Cyclic voltammograms of electrochemical aptamer-based (EAB) sensors recorded before and after regeneration and storage stability tests. (A) Planar EAB sensor before and after 10 regeneration cycles. (B) MSE EAB sensor before and after 10 regeneration cycles. (C) Planar EAB sensor before and after 14 days of storage in PBS at 4 °C. (D) MSE EAB sensor before and after 14 days of storage in PBS at 4 °C. All measurements were performed in artificial sweat solution by cycling the electrode potential between 0 and –0.5 V versus Ag/AgCl reference electrode at a scan rate of 0.1 V·s^{-1}.

Reference:

[1] S. Komorsky-Lovric and M. Lovri, *Electrochimica Acta*, Vol. 40, No. 11, pp. 1781-1784, 1995.
